# Plasma Peptide Biomarker Discovery for Amyotrophic Lateral Sclerosis by MALDI –TOF Mass Spectrometry Profiling

**DOI:** 10.1371/journal.pone.0079733

**Published:** 2013-11-05

**Authors:** Laurence Conraux, Catherine Pech, Halim Guerraoui, Denis Loyaux, Pascual Ferrara, Jean-Claude Guillemot, Vincent Meininger, Pierre-François Pradat, François Salachas, Gaëlle Bruneteau, Nadine Le Forestier, Lucette Lacomblez

**Affiliations:** 1 Exploratory Unit, Sanofi, Toulouse, France; 2 Centre référent maladies Rares, APHP, UPMC, La Salpêtrière Hospital, Paris, France; University of Florida, United States of America

## Abstract

The diagnostic of Amyotrophic lateral sclerosis (ALS) remains based on clinical and neurophysiological observations. The actual delay between the onset of the symptoms and diagnosis is about 1 year, preventing early inclusion of patients into clinical trials and early care of the disease. Therefore, finding biomarkers with high sensitivity and specificity remains urgent. In our study, we looked for peptide biomarkers in plasma samples using reverse phase magnetic beads (C18 and C8) and MALDI-TOF mass spectrometry analysis. From a set of ALS patients (n=30) and healthy age-matched controls (n=30), C18- or C8-SVM-based models for ALS diagnostic were constructed on the base of the minimum of the most discriminant peaks. These two SVM-based models end up in excellent separations between the 2 groups of patients (recognition capability overall classes > 97%) and classify blinded samples (10 ALS and 10 healthy age-matched controls) with very high sensitivities and specificities (>90%). Some of these discriminant peaks have been identified by Mass Spectrometry (MS) analyses and correspond to (or are fragments of) major plasma proteins, partly linked to the blood coagulation.

## Introduction

Amyotrophic Lateral Sclerosis (ALS) is the most common motor neuron disease (worldwide prevalence: 4/100 000), leading to increasing muscle weakness and muscle atrophy. The expected lifetime after diagnosis is 3-5 years (prognosis is worse for bulbar-onset patients). The important heterogeneity of the disease leads to consider ALS as a syndrome rather than a specific disease [1]. Considering this heterogeneity and the importance of an early diagnosis both for clinical trials and clinical daily practice, there is a major demand for biomarkers of the disease. Identification of specific biomarkers of ALS could enable early detection, diagnosis and prognosis, and could be helpful to evaluate the efficacy of new treatments, e.g. testing of new therapies in clinical trials.

Numerous proteins within the CSF or serum/plasma, two body fluids which are promising sources of biomarkers, have been suggested as potential ALS biomarkers including hormones and growth factors (Insulin, EPO, FGF-2PEDF…); inflammatory system related (Caspase-1, RANTES, TNF…); neuron specific (NFL); enzymes and enzyme inhibitors (CysC, Angiogenin…) and others proteins (VGF, TTR, Aβ42…) [2-6]. However, either these candidate biomarkers have not proved enough specificity and sensitivity, or have not been yet enough validated to be used as a routine test for identifying subjects at risk for developing the disease or predicting outcomes following treatment. In this perspective, combination of several biomarkers may significantly increase the specificity and accuracy of ALS diagnosis [7].

Recent studies have established that distinctive plasma/serum peptide patterns with clinical relevant outcomes can be obtain through Matrix Assisted Laser Desorption / Ionisation Time-Of-Flight (MALDI-TOF) mass spectrometry after peptide capture and enrichment using grafted phase onto magnetic particles [8-10]. With this approach, a limited subset of peptides (peptide signature) could provide class discrimination between patients and healthy controls. Recently, this approach has been applied with success to classify ALS patients versus age-matched controls based on CSF biomarker panel [11]. However, a diagnosis based on a simple blood test would be easier to implement. Less complex and invasive than the lumbar puncture, the blood sampling is also easily accepted by the patients. 

With this aim, plasma biomarker discovery for ALS diagnostic was undertaken using C8 or C18-reverse phase magnetic beads and MALDI-TOF mass spectrometry analysis (read-out). In a first step, biomarker discovery was realized within a training set of plasma samples from 30 patients with ALS and 30 healthy age-matched controls. Then, predictive models were tested using a separate test set of 10 ALS patients and 10 control subjects. Considering the limited number of patients included in our study, this process of biomarker discovery and validation was repeated ten times using each time new randomly generated sets. By so doing, the minimum number of peaks necessary to classify blinded samples with high sensitivity and specificity was determined. The identification of these biomarker candidates were then carried out using liquid chromatographic separation coupled to mass spectrometry. 

## Materials and Methods

### ALS patients and controls

Patients with definite, probable or laboratory probable ALS and age-matched healthy controls were recruited in the National Referral Center at La Salpêtrière Hospital, Paris, France. A total of 80 plasma samples were included in this study: 40 from patients with ALS (32 spinal / 8 bulbar) and 40 healthy volunteers, matched by age and sex. The demographic features of all ALS patients and healthy controls are shown in table 1. As the age distribution of both groups of patients was significantly different, one can indeed not fully exclude that our data was biased by this offset in age. However, no relevant correlation between the intensity of the MALDI peak and the respective age of the patients has been found (data not shown). It is on this basis that we have taken all patients from both groups into account.

**Table 1:** Demographics of ALS patients and control subjects.

		**ALS Patients (n=40)**	**Healthy controls (n=40)**
**Age (yrs)**	Mean ± SD	57.8* ± 12.5*	50.9 ± 9.3
	Min:Max	36:81	29:73
**Sexe**	Male	25 (62.5%)	25 (62.5%)
	Female	15 (37.5%)	15 (37.5%)
**Race**	Caucasian / white	38 (95%)	38 (95%)
	Black	0	2 (5%)
	Other	2 (5%)	0
**Weight (kg)**	Mean ± SD	69.40 ± 10.37	71.32 ± 11.74
	Min:Max	46.0 : 88.0	43.4 : 97.0
**BMI (kg/m^2^)**	Mean ± SD	24.05 ± 3.59	24.42 ± 3.04
	Min:Max	18.4 : 36.6	17.2 : 30.3

Uncompleted matching in age & race due to high difficulties in recruitment of control subjects.

Statistical analysis was performed by t-test assuming unequal variances (Bartlett's test), * p<0.05

The average time of disease duration for ALS patients at the time of plasma sampling was 2.4 years with a standard deviation of 1.7 years. All ALS patients received concomitant medications during the study except one (Patient Nb 250002016). The diagnosis of ALS in this patient remains uncertain despite several clinical examinations. Biomarker research step was done with 30 ALS samples and 30 healthy control samples (training set). Remaining samples are used for validation step (blinded testing set, 10 samples per group). Sample distribution was carried out randomly. The protocol was submitted to an independent ethics committee for review and written approval (CPP – Ile-de-France VI, for Comité de Protection des Personnes). All participants provided their written consent in this study as approved by the ethical committee.This Clinical was conducted in accordance with the principles laid down by the 18th World Medical Assembly (Helsinki, 1964) and all applicable amendments laid down by the World Medical Assemblies and the ICH guidelines for Good Clinical Practice (GCP).

### Reagents

Superparamagnetic, silica-based particles, surface-derivatized with common reversed-phase ligands (C8 or C18), were obtained from Bruker Daltonics (purification kit MB-HIC 8) or Dynabeads (RCP 18). Acetonitrille (ACN), ethanol, acetone and trifluoroacetic acid (TFA) were purchased from Sigma-Aldrich. Recrystallized a-cyano-4-hydroxycinnamic acid (CHCA) was purchased from LaserBio Labs (France).

### Biological Samples

Intravenous blood samples were collected in EDTA containing tubes (BD diagnostic spray K2-EDTA), inverted to mix, and centrifuged within 2 hours after sample collection (1,300g for 15 min) at room temperature. As soon as the centrifugation was completed, the plasma was collected by maintaining the pipette tip in the middle region of the liquid portion, making sure not to aspirate too near the top surface of the cell pellet. All collected plasma were aliquoted into polypropylene screw-capped tubes (AB gene SMART SCAN RACKS) and immediately frozen at -80°C. Samples were randomized prior splitting into aliquots in 96-well-plates (20µl/well). A pre-defined number of commercially available sera was placed in the same rack for tracking the accuracies of sample preparation (S-7023, Sigma). The 96-well-plates were stored at -80°C until use.

### Magnetic bead-based sample preparation and MALDI-TOF MS-profiling

The extraction protocols provided with the magnetic beads are implemented in an automat (ClinProt Robot, Bruker), according to the manufacturer's protocols, except for the extraction step: 10μl acetonitrile/water 30/70. The volume of native plasma used was adjusted to the type of beads, 5 or 20µl of native plasma were added to 5µl of MB-C8 or RCP 18 beads respectively. Three independent experiments were done for each plasma sample, with each type of beads (C8 and C18). The eluates were transferred to another 96 well plate and immediately spotted on a MALDI plate. 0.3 μl of the peptide extract was loaded onto the MALDI target with 0.3 μl of fresh matrix solution (5 mg/ml CHCA, LaserBio Labs, ethanol/acetone 2/1) and left to dry at room temperature. For each extract, three deposits were realized (MapII robot, Bruker). MALDI spectra were recorded using a Perseptive Voyager-DE STR instrument (AB Sciex) equipped with a 20 Hz nitrogen laser (337nm) and operated in delayed extraction linear mode with a acceleration voltage of 20 kV. An external calibration using PepMix3 (LaserBio Labs) was carried out before data acquisitions. For each spectrum (1-10 kDa), 600 laser shots at different locations were summed. Using these replications, 9 spectra were acquired per sample and exported as ASCII files.

### Spectra Treatment and Statistical Analysis

ASCII files from Voyager DE-STR (data available on request to the corresponding author) were treated inside of R environment for baseline subtraction and conversion of the dataset to .csv files readable by ClinProTools (Bruker), the software used for data analysis. The files were processed for normalization of spectra; recalibration and average peak list calculation (signal/noise ratio ≥ 3 on the class averaged). Quantifications of these peaks in each spectra are stored in a matrix which is used by the program or exported for external statistical analysis (Table S1 and S2). Principal component analyses (PCA) from exported peak matrix were performed with Spotfire Decision Site V8 packages and peak discrimination analyses were done with DAnTE [12].

Predictive models for classification of unknown samples were generated using a Support Vector Machine-based procedure (SVM) available in the ClinProTools software (Bruker). This SVM-based procedure is an algorithm relying on two statistical analysis methods, SVM and the k-nearest neighbour (kNN) method. To estimate the prediction capability for unknown samples, we used a standard cross-validation technique with a training set consisting of randomly chosen subsets containing 90% of each class; the remaining 10% of the samples from each class were left as test sets. Accuracy was estimated over 10 consecutive runs. The number of neighbours for the KNN classification is fixed to 5 and the final number of peaks in the model is either free or limited depending of the approach selected. These generated SVM-based models were then tested against the validation set.

### Peptide identification

The identification of biomarkers was done using a specific coupling Nano-LC/Probot/FTICR-MS. The liquid chromatographic (LC) separation was performed on a Ultimate (Dionex, Sunnyvale, USA) nanoflow system connected to a μ-Tee to split the flow between the Micro Fraction Collector (Probot System, Dionex, Sunnyvale, USA) and a 9.4T ApexQ III FTICR mass spectrometer (Bruker Daltonics, Billerica, MA, USA). 60 μl of the peptide extract filtered on spin filter of 0.22 μm were loaded on-line and pre-concentrated on the μ-precolumn (0.3mm I.D/ X 5mm, C8 Zobrax 300SB, Agilent) at a flow rate of 20 μl/min with 0.2% formic acid in water during 5 min. Peptides were eluted on a reverse-phase column (C18 Acclaim 300 A, 150 mm X 75μm I.D of 5um of particle size) with a linear gradient of 5-50% solvent B (H2O/CH3CN/HCOOH, 10:90:0.2, by vol.) for 95 min, 50-95% solvent B for 5 min at a flow rate of 350 nl/min. The FTICR mass spectrometer was operated in full scan MS spectra (from m/z 400-2000) with 512K data points. The data were calibrated internally using previously identified peptides. The Probot Micro Fraction Collector was operated in deposit mode, onto a MALDI target, each 12 sec during all the LC run. Each deposit corresponds to a mixture of the flow from the NanoLC and a matrix solution (5 mg/ml CHCA, LaserBio Labs in CH3CN/H2O/TFA, 70:30:0.2, by vol.) at a flow of 600 nl/min through a μ-Tee. One MALDI spectrum was acquired per HPLC spotting if a signal was detected (minimum intensity 2000 with signal to noise ration > 5). LC-MALDI acquisitions were done using a Perceptive Voyager-DESTR instrument (Applied Biosystems) equipped with a 20 Hz nitrogen laser (337nm) and operated in delayed extraction linear mode (300nsec) with an acceleration voltage of 25 kV and a grid voltage of 93%. An external calibration using PepMix3 (LaserBio Labs) was carried out before sample acquisitions. For each spectrum (1-10 kDa), 300 laser shots at different locations were summed (50 scans per position). FTMS and MALDI mapping: the list of all the detected peptides (m/z) and their corresponding retention time was extracted from the LC/FTICR-MS runs analyzed using an in-house software (DIFFTAL, software tools developed in Sanofi under MATLAB® environment for label-free differential analysis of complex proteomic mixture and dedicated to high resolution LC/MS).This list was then matched with the LC/MALDI data for assign an accurate masse (up to 3ppm) to each peptide of interest. RAW data are available on request to the corresponding authors.

### NanoLC-MS/MS analysis

Biomarker identifications were performed through LC/MS/MS experiments on an LTQ Orbitrap mass spectrometer (Thermo Fisher, USA) equipped with a nanoelectrospray source. LC conditions were the same as in the Nano-LC/Probot/FTICR-MS setup. The mass spectrometer was operated in the data-dependent mode to automatically switch between Orbitrap-MS and Orbitrap-MS/MS acquisition for each biomarker of the included list (characterized by their m/z and RT). Survey full scan MS spectra (from m/z 300-1700) were acquired in the Orbitrap with a resolution of 60,000 at m/z 400. The AGC was set to 1x10^6^ with a maximum injection time of 500ms. Each ion of the list was then isolated for fragmentation in the LTQ linear ion trap using a normalized collision energy of 35% at the default activation q of 0.25 with an AGC settings of 1 × 10^5^ and a maximum injection time of 100 ms. Resulting fragments were recorded in the Orbitrap with a resolution of 30 000 at m/z 400.

### LC-MS/MS data Processing

Each LC-MS/MS data acquired using the Xcalibur software (version 2.07, Thermo-Fisher Scientific) was deconvoluted to charge state 1 using the software DIFFTAL in order to create the MS/MS peak list (MGF file) which is used for database searching. 

### Database searching

Database searches were done using an internal MASCOT server (version 2.1, matrix Science; http://www.matrixscience.com/) using the NCBInr database containing 534242 entries. The search parameters used for post-translational modifications were dynamic modifications of +15.99491 on methionine residues (oxidation), of +42.010565 on protein N-terminal residues (N-terminal acetylation) and -17.026549 on N-terminal glutamine residues (N-Pyroglu). The precursor mass tolerance was set to 10 ppm and the fragment ion tolerance was set to 0.03 Da with no enzyme restraint. Scaffold (version Scaffold_4.0.5, Proteome Software Inc., Portland, OR) was used to validate MS/MS based peptide. Peptide identifications were accepted if they could be established at greater than 90.0% probability to achieve an FDR less than 0.1% by the Scaffold Local FDR algorithm assigned by the Protein Prophet algorithm [13].

## Results

### Unsupervised analyses of peptide signals from MALDI-based plasma profiling differentiate ALS patients and healthy volunteers

**Figure 1:**
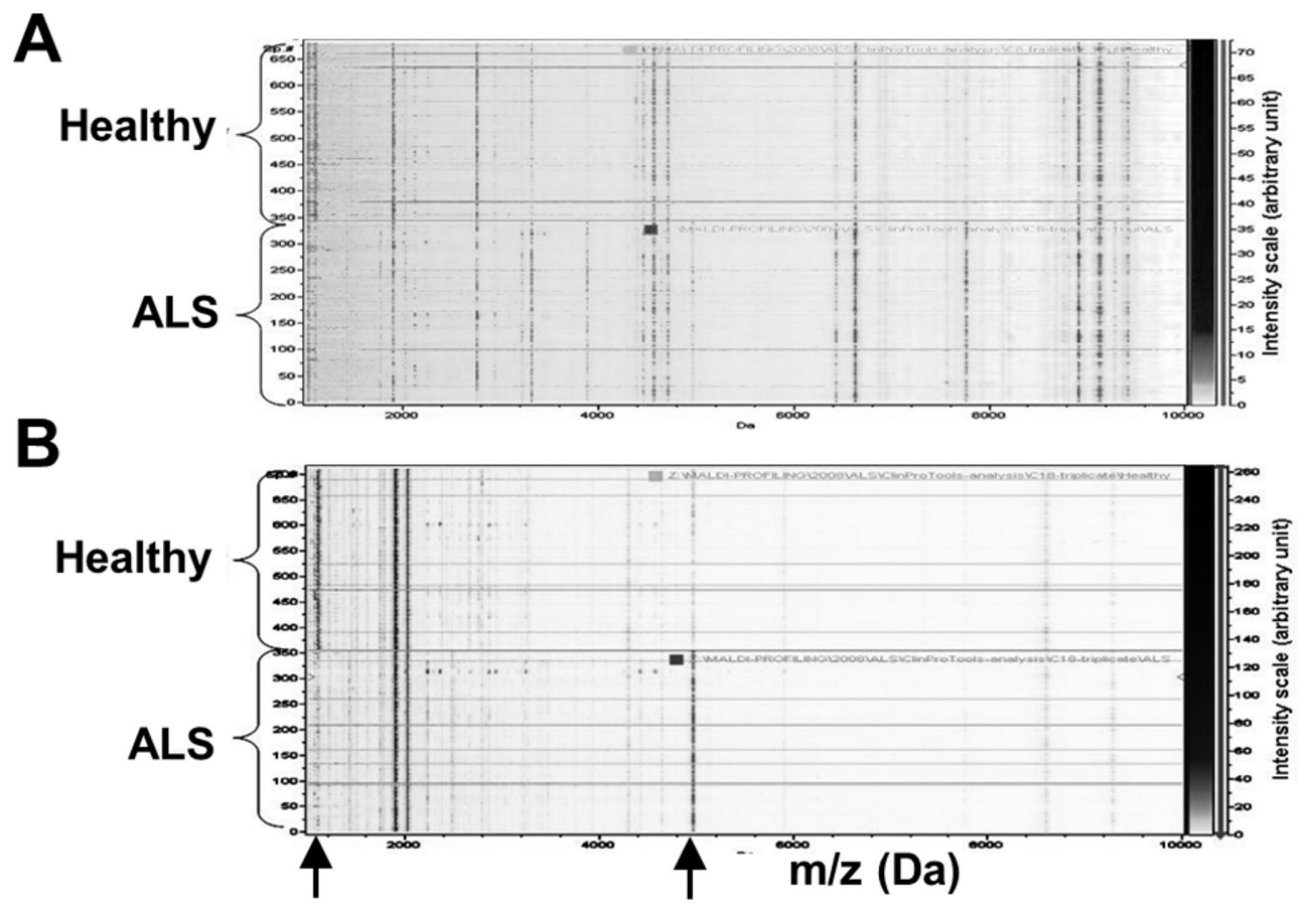
MALDI-TOF profiles representation. 2D C8 (A) and C18 (B) MALDI-TOF profiles representation (Log_10_ Intensity with results expressed in arbitrary units) of ALS (bottom) and healthy (top) patients; Mass range (x-axis): 1-10kDa, underlined spectra correspond to excluded data (miss-calibrated spectrum).

**Figure 2:**
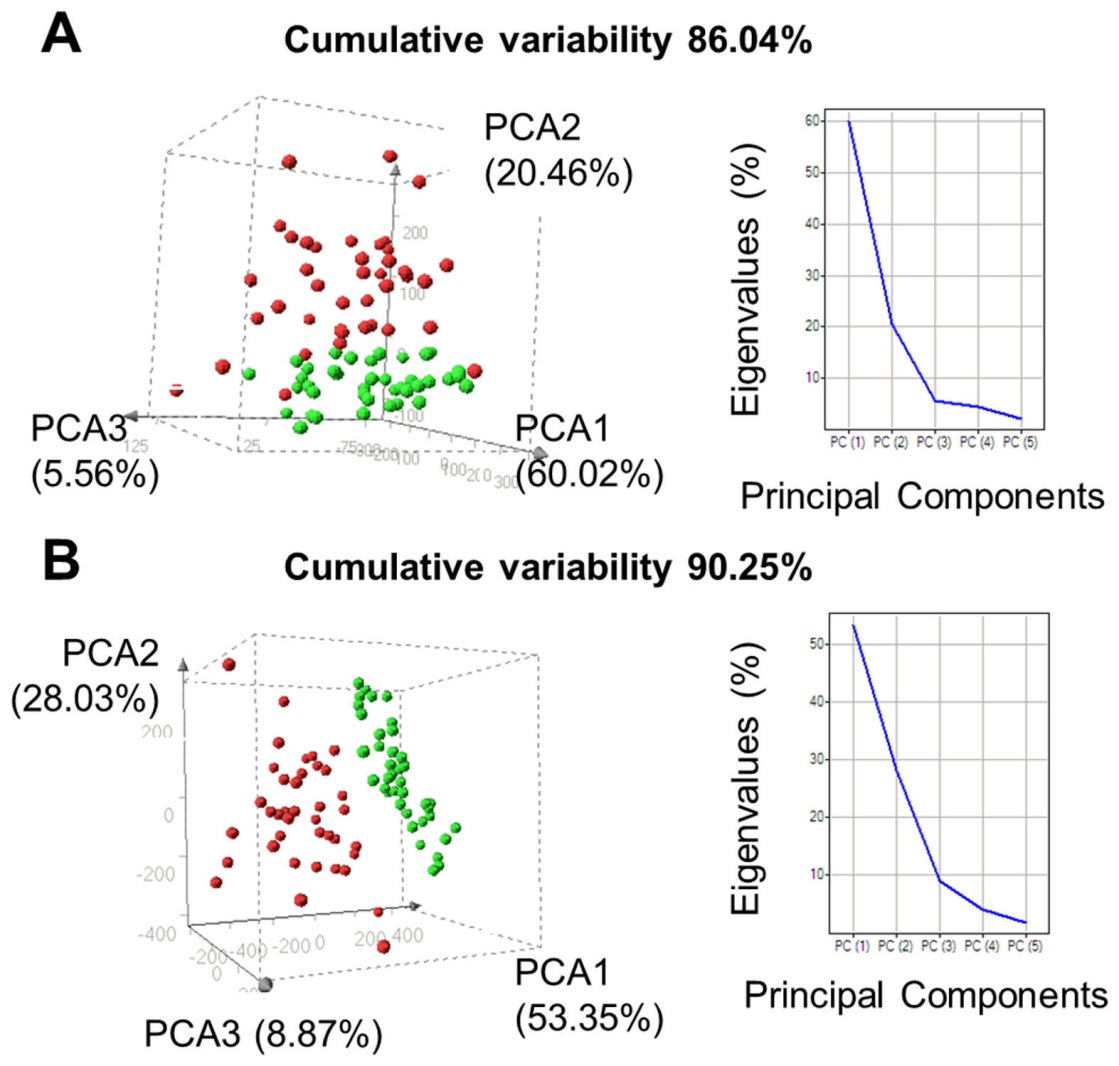
Principal component analysis. The first 3 principal components which account for most of the variance in the original data set are shown A) from MS-data of C8-beads sample preparation and B) from MS-data of C18-beads sample preparation; ALS patients (red) and healthy controls (green) ; 1 dot per patient (average of 9 spectra per patient); Eigenvalues screen plots are at the right of each PCA.

We analyzed the plasma peptide profiles of 40 ALS patients and 40 healthy volunteers. All 80 plasma samples were processed fully automatically as a single batch and with automated MALDI-TOF MS analysis. After normalization and alignment of the all processed spectra, 158 unique peaks, with a signal to noise threshold equal or greater than 3 on the average spectrum, could be detected in the C18 dataset and 131 unique peaks in the C8 dataset. MALDI profiles are displayed on Figure 1. Matrixes containing the normalized intensities of all detected peaks from peptide extracted on magnetic beads coated with C8 or C18 phase for each of the samples (9 spectra per patients) were then used for unsupervised statistical analyses using principal component analysis (PCA). Two separate point clouds corresponding each to one class of samples, ALS patients and healthy controls, are clearly highlighted by these analyses (Figure 2).

### Selection of peptide ion signatures to separate ALS patients from the healthy volunteers

**Figure 3:**
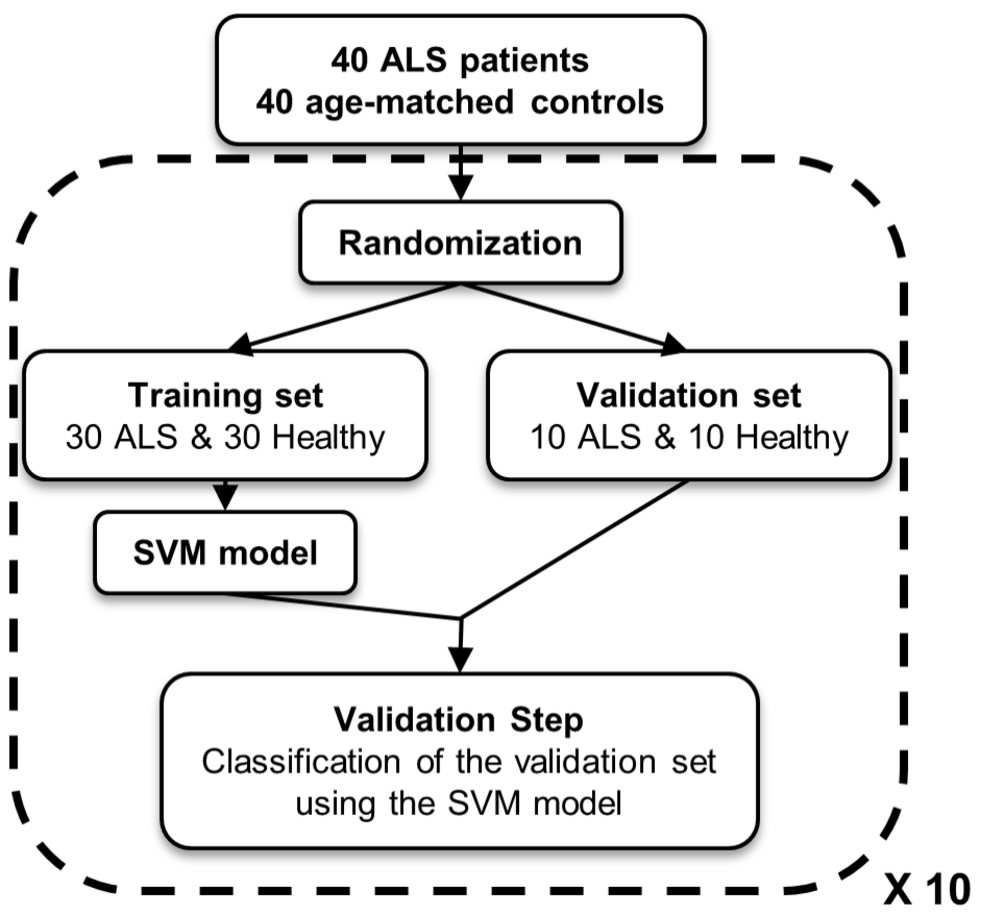
Workflow of the SVM model generation. Data were randomly separated into two data sets, a training set (30 ALS patients and 30 healthy controls) used for the biomarker discovery step and a validation set used for classification of blinded samples (10 ALS patients and 10 healthy controls). This process of data randomization, discovery and validation was repeated ten times.

**Figure 4:**
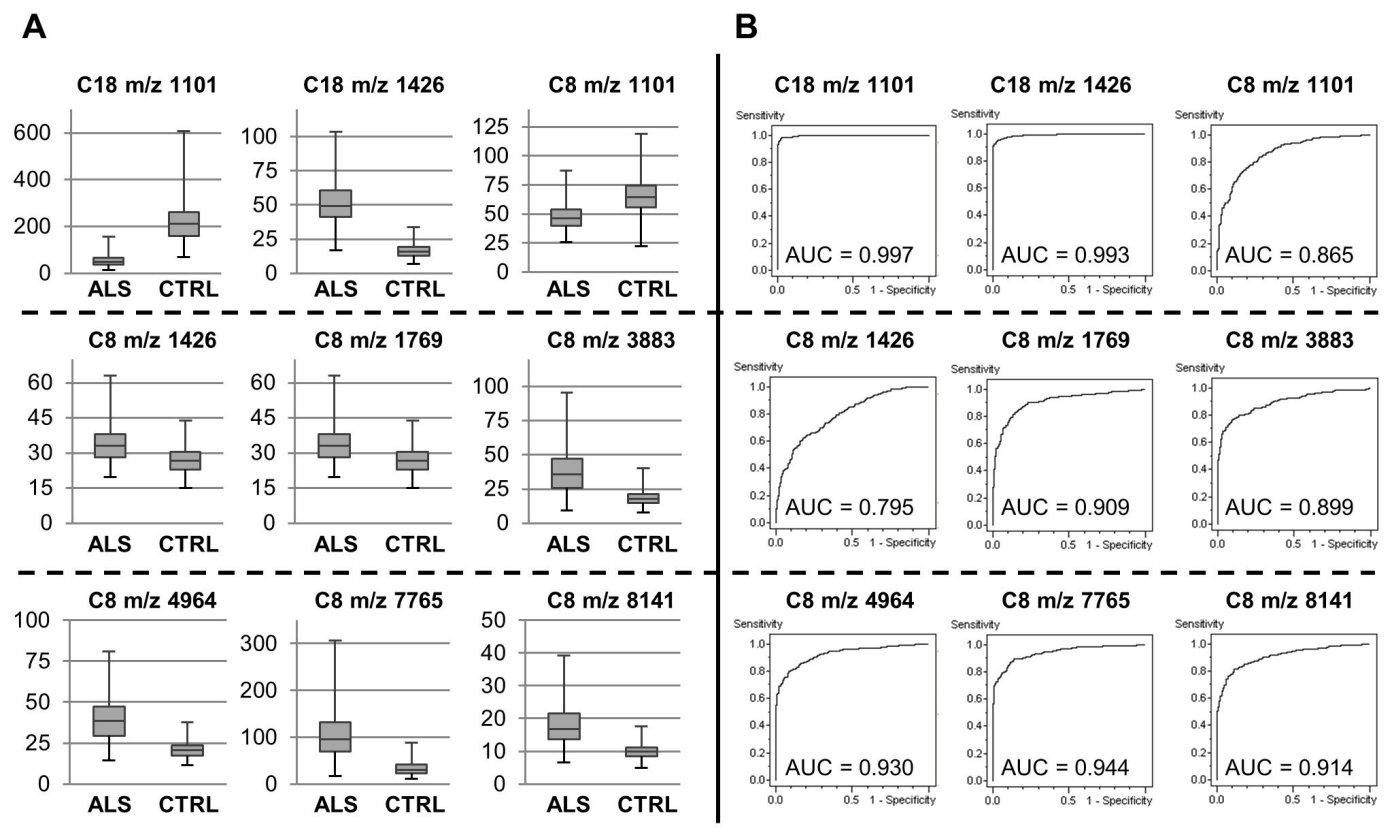
Peak variance and ROC curves. A/ Representation of the expression level for some of the most discriminating C8- or C18-peaks (x-axis, peak intensity), B/ corresponding receiver operating characteristic curve (ROC) with ALS group define as positive class.

Developing a clinical diagnostic involving more than a dozen signals is not realistic. Therefore, we sought to select the most discriminating peaks to develop predictive models based on a minimum number of signals. To overcome the limited number of patients included in our study, data were randomly separated into two data sets, one for the discovery step (training set, with 30 ALS patients and 30 healthy controls) and the second for the validation (classification of blinded samples, 10 ALS patients and 10 healthy controls); and this process was repeated 10 times (Figure 3). From each of the 10 generated training sets, predictive models were calculated using a supervised method based on support vector machine (SVM). In this process, automatic detection was selected for determining the best number of peaks to be integrated in a model. To access the ability of each individual SVM-based model to correctly classify blinded samples, the corresponding validation sets were then used (The SVM model n°1 against the validation set n°1 and so on). Results are summarized in tables 2 and 3. From C18 data, the number of selected signal changes from 2 to 21 according to the model while for C8 data the number of selected signal changes from 7 to 23 (Table 2). Whichever the SVM-based model, the cross validation values and the recognition capacity are very high; at least greater than 94% (Table 3). What is more, the values of sensitivity and specificity obtained from the classification of the validation data sets are also high. C18 SVM-based models correctly classify all the spectra of the control group and at least 89% of the spectra from ALS group. Results with C8 SVM-based models are similar even although the values are a little less good with 68.8% and 90.5% of well classified spectra for ALS and control groups respectively. Under these results, the minimum of peaks for well classified data is at least 2 peaks for C18-samples (1101 and 1426) and 7 peaks for C8-samples (1101, 1426, 1769, 3883, 4964, 7765 and 8141). The high discriminant power of some C18- or C8-peaks is also highlighted by the high p-values obtained from the analysis of the peak variance (ANOVA) using log-2 transformed data (Table S3). 69.5% of the C8-peaks and 86% of the C18-peaks show p-value <0.05; with 1/3 of the peaks that are ALS up-regulated signals. Only one peak has a 2-fold change among C8-signals (peak 7765) while 14 signals pass this fold change value among C18-data (10 ALS up- and 4 down-regulated: 1426, 2230, 2483, 2511, 3469, 4426, 4920, 4979, 4964, 5004 and 1079, 1101, 1127, 2755). A comparison between this ANOVA test and the selected peaks over all the SVM-based models shows that, as expected, SVM models are based on the most discriminating signals. The expression levels of some of the most discriminating peaks are presented in Figure 4 with the corresponding receiver operating characteristic curves.

**Table 2:** List of the selected signals for the SVM-based models.

**C8 SVM-based models**													**C18 SVM-based models**											
**m/z**	**1**	**2**	**3**	**4**	**5**	**6**	**7**	**8**	**9**	**10**	*		m/z	**1**	**2**	**3**	**4**	**5**	**6**	**7**	**8**	**9**	**10**	*
**7765**	x	x	x	x	x	x	x	x	x	x	10		1101	x	x	x	x	x	x	x	x	x	x	10
**4964**	x	x	x	x	x	x	x	x	x	x	10		1426	x	x	x	x	x	x	x	x	x	x	10
**8141**	x	x	x	x	x	x	x	x	x	x	10		1127	x	x	x	x	x	x	x	x		x	9
**1769**	x	x	x	x	x	x	x	x	x	x	10		2511	x	x	x	x	x	x	x	x		x	9
**3883**	x	x	x	x	x	x	x	x	x	x	10		4964	x	x	x	x	x	x	x	x		x	9
**1101**	x	x	x	x	x	x	x	x	x	x	10		1769	x	x	x	x		x	x	x		x	8
**1426**	x	x	x	x	x	x	x	x	x	x	10		2483		x	x	x	x	x	x	x		x	8
**9288**	x	x		x	x	x	x	x		x	8		4979	x	x	x	x		x	x			x	7
**6630**	x	x		x	x	x	x	x			7		3218			x	x	x	x	x	x			6
**3316**		x	x	x	x		x	x			6		1742			x	x	x	x	x				5
**6432**	x			x	x	x	x	x			6		2194		x		x		x	x			x	5
**4644**	x	x		x	x	x					5		2662		x	x	x		x				x	5
**7345**	x	x		x	x	x					5		4920		x	x	x		x				x	5
**2793**	x	x			x		x	x			5		5004		x	x	x		x				x	5
**7839**		x		x		x		x			4		3469			x	x		x	x				4
**6588**	x			x	x		x				4		1085			x				x			x	3
**5042**	x				x	x		x			4		1937			x			x	x				3
**1898**				x	x	x					3		2755		x		x			x				3
**1881**	x	x				x					3		5109		x	x							x	3
**1382**					x	x		x			3		1784		x	x								2
**3217**	x					x					2		2770				x		x					2
**4343**		x		x							2		2792		x		x							2
**8765**	x	x									2		3366		x	x								2
**4102**	x					x					2		3405			x	x							2
**9485**		x						x			2		3762			x			x					2
**2024**	x					x					2		1452				x							1
**1127**					x						1		2230						x					1
**7639**					x						1													
**9379**		x									1													
**4282**					x						1													
**7469**						x					1													
**9935**					x						1													
**8685**					x						1													
**6940**								x			1													
**3087**						x					1													
**4856**				x							1													

* Number of time which a peak was present

**Table 3:** Statistical data for each SVM-based model.

**C18 - Model Nb**	**1**	**2**	**3**	**4**	**5**	**6**	**7**	**8**	**9**	**10**		

**Nb of signals**	**7**	**17**	**21**	**20**	**8**	**19**	**15**	**8**	**2**	**14**	**Avg**	**StDv**
Cross Validation (%) *	99.2	98.8	98.6	99.6	99.8	99.8	99.8	99.8	98.1	98.6	99.20	0.65
Recognition (%) *	99.8	100	100	100	100	99.6	100	99.6	99.8	100	99.88	0.17
**Sensitivity (%)**	100	97.6	100	100	97.7	89.2	98.9	100	97.5	97.6	97.85	3.24
**Specificity (%)**	100	100	100	100	100	100	100	96.6	100	100	99.66	1.08

**C8 - Model Nb**	**1**	**2**	**3**	**4**	**5**	**6**	**7**	**8**	**9**	**10**		

**Nb of signals**	**20**	**19**	**8**	**18**	**23**	**22**	**13**	**18**	**7**	**8**	**Avg**	**StDv**
Cross Validation (%) *	95.6	96.8	94.2	95.4	93.1	94.0	94.2	93.9	95.7	94.3	94.70	1.12
Recognition (%) *	97.9	98.8	97.1	98.9	98.2	96.6	96.9	98.4	98.2	97.3	97.83	0.79
**Sensitivity (%)**	89.0	80.2	92.3	68.8	89.7	97.4	93.5	88.9	83.3	95.1	87.82	8.46
**Specificity (%)**	90.5	100	95.0	98.7	98.7	94.4	97.3	97.5	100	97.3	96.94	2.93

* overall

Using the minimum of common peaks previously identified by unsupervised analyses, 2 for C18 data (m/z 1101 and 1426) and 7 for C8 data (m/z 1101, 1426, 1769, 3883, 4964, 7765 and 8141, Table 2), new SVM-based models were generated using the 10 previously defined training sets. Each of these new models was able to classify its corresponding set with very high sensitivity and specificity (Table 4). The sensitivity and specificity obtained from each SVM-based models stemming from C18 data are all at least equal to 98% and virtually identical. By comparison, the values for SVM-based models stemming from C8 data are somewhat weaker and less homogeneous, in particular for the sensitivity. However, the values are still high with a minimum of 79% and 96% for the sensitivity and specificity respectively (average values, 90% and 98% respectively). 

**Table 4:** SVM-based models generated with a restricted number of peaks.

**C18 - Models**												
Exp	**1**	**2**	**3**	**4**	**5**	**6**	**7**	**8**	**9**	**10**	**Avg**	**Stdev**
**Peak weight**												
1101	1.18	1.22	1.11	1.23	1.32	1.34	1.27	1.32	1.21	1.03	1.22	0.10
1426	0.76	0.90	0.83	0.82	0.85	0.89	0.78	0.82	0.87	0.85	0.84	0.04
**Cross Validation**												
Overall:	**0.99**	**0.98**	**0.99**	**0.99**	**0.99**	**0.99**	**0.99**	**0.99**	**1**	**0.99**	**0.99**	>0.01
Class 1	0.99	0.98	0.99	0.99	1.00	0.99	0.99	0.99	1	0.98	0.99	0.01
Class 2	1	0.99	1	0.99	0.99	1	1	0.99	1	0.99	0.99	>0.01
**Recognition Capability**												
Overall:	**0.99**	**0.99**	**0.99**	**0.99**	**1**	**0.99**	**0.99**	**0.99**	**1**	**0.99**	**0.99**	>0.01
Class 1	0.99	0.99	0.99	0.99	1	0.99	0.99	0.99	1.00	0.99	0.99	>0.01
Class 2	1	1	0.99	1	1	1	1	1	1	0.99	1	>0.01
**Validation (Set 2)**												
ALS	1	0.98	0.99	1	0.98	1	1	0.99	0.98	1	0.99	0.01
CTRL	1	1.00	0.99	0.99	1	0.98	0.99	1	1	0.99	0.99	0.01

**C8 - Models**												
Exp	**1**	**2**	**3**	**4**	**5**	**6**	**7**	**8**	**9**	**10**	**Avg**	**Stdev**
**Peak weight**												
1101	0.80	0.55	0.74	0.60	0.69	0.73	0.74	0.75	0.56	0.76	0.69	0.09
1426	0.73	0.58	0.70	0.50	0.38	0.42	0.52	0.47	0.58	0.57	0.54	0.11
1769	0.94	0.89	0.96	0.94	1.00	0.95	0.93	1.00	1.04	1.03	0.97	0.05
3883	0.52	0.61	0.46	0.58	0.45	0.49	0.48	0.62	0.57	0.47	0.52	0.06
4964	0.88	0.91	0.96	0.84	0.82	1.02	0.96	0.83	0.81	0.85	0.89	0.07
7765	0.58	0.67	0.49	0.81	0.63	0.53	0.59	0.67	0.70	0.59	0.63	0.09
8141	0.53	0.60	0.52	0.59	0.56	0.34	0.53	0.61	0.67	0.61	0.56	0.09
**Cross Validation**												
Overall:	**0.93**	**0.97**	**0.93**	**0.97**	**0.93**	**0.94**	**0.95**	**0.96**	**0.97**	**0.95**	**0.95**	**0.02**
Class 1	0.89	0.97	0.90	0.94	0.87	0.90	0.94	0.92	0.93	0.93	0.92	0.03
Class 2	0.98	0.98	0.95	1.00	0.99	0.97	0.97	1	1	0.97	0.98	0.02
**Recognition Capability**												
Overall:	**0.96**	**0.98**	**0.96**	**0.98**	**0.97**	**0.96**	**0.97**	**0.97**	**0.98**	**0.96**	**0.97**	**0.01**
Class 1	0.94	0.97	0.94	0.97	0.94	0.92	0.94	0.95	0.96	0.94	0.95	0.01
Class 2	0.99	1	0.98	1	1	0.99	0.99	0.99	1	0.99	0.99	0.01
**Validation (Set 2)**												
ALS	0.94	0.79	0.91	0.79	0.94	1	0.92	0.91	0.83	0.95	0.90	0.07
CTRL	1	1	0.99	0.98	0.97	0.99	0.96	0.96	1	0.99	0.98	0.02

For C18 models two peaks were used and for C8 models 7 peaks were used. For each set of data (Experiment 1 to 10) the weight of each peak in the SVM-based model is specified. Cross validation values as well Recognition values are listed just below; then sensitivity and specificity from classification of the validation set (at level spectra) are indicated, class1 = ALS and class 2 = control group (CTRL)

### Data analysis depending on the type of ALS or the gender of the patients

**Figure 5:**
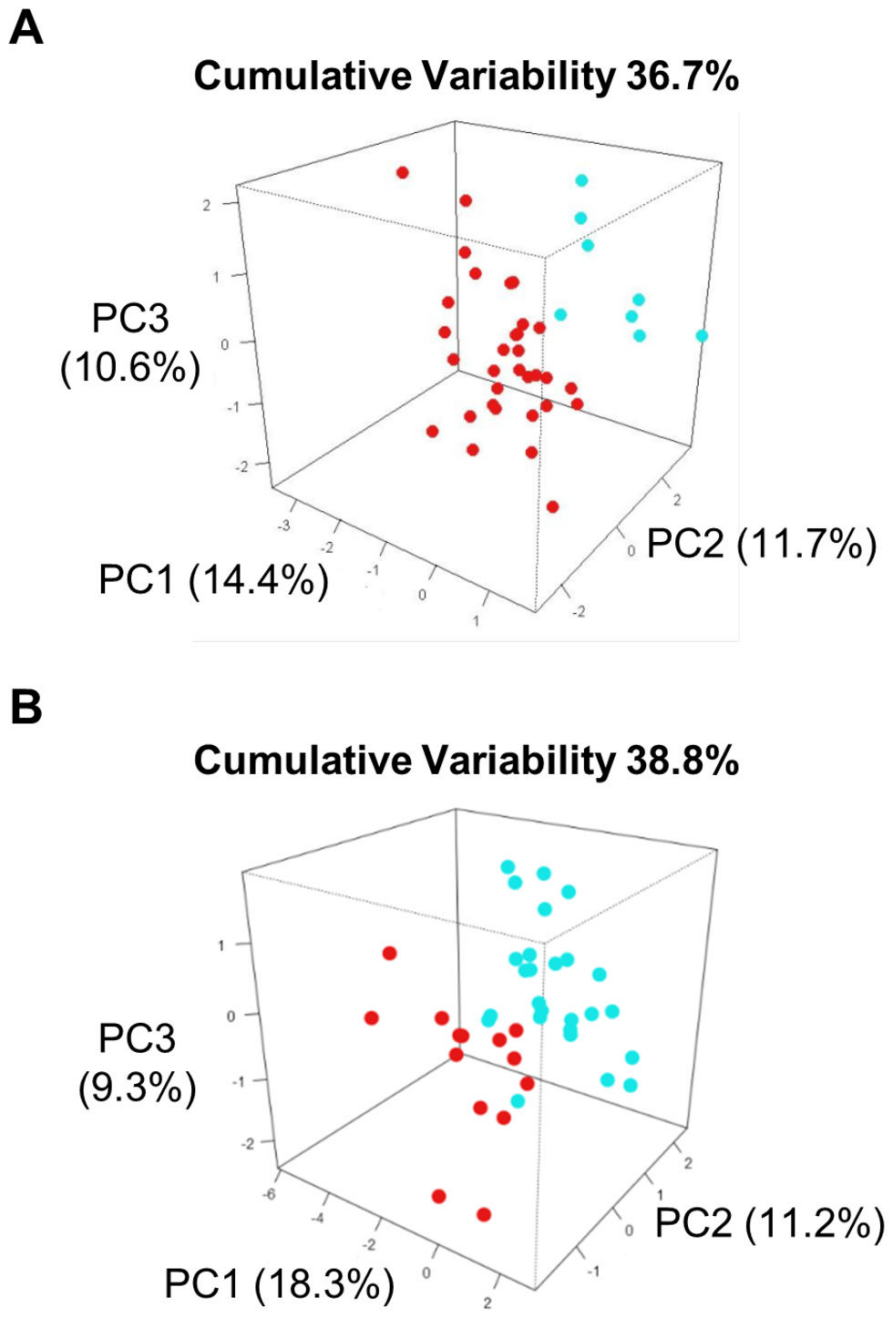
PLS results. Analysis of C18 data from ALS patients A/ spinal-onset (red) and bulbar-onset (turquoise); B/ female (red) and male (turquoise) - Each dot correspond to one patient (average of 9 spectra per patient).

In this study, 32 ALS patients had a spinal onset (10 women / 22 men) and 8 a bulbar onset (4 women / 4 men). To determine if some peaks are significant signals to discriminate between ALS subtypes, an orientated statistical analysis of C18 MALDI data was done. PLS (Partial Least Square) analysis clearly shows that spinal and bulbar ALS patient can be differentiated on the base of the C18 MALDI data (Figure 5.A). In the same way, the PLS analysis based on the gender shows that C18 MALDI profiles can also allow the ALS patients classification depending on the gender (Figure 5.B). However, implicated signals (p-value<0.05) in gender differentiation do not match with the list of significant peaks that lead to the ALS subtype differentiation (Table 5). On the other hand, only one peak selected as gender discriminative signal appears in 5 of the 10 SVM-based models defined for ALS diagnostic during the discovery step (Table 2, peak 5004). However, as this peak was not present in all of the 10 SVM-based models for ALS diagnostic, it has not been selected for the final model.

**Table 5:** Discriminating peaks between spinal and bulbar subtypes or gender of ALS patients.

**Spinal/bulbar subtypes**			**Gender**	
**m/z**	**p-value**		**m/z**	**p-value**
**8052**	4.00E-03		**5905**	2.00E-04
**7499**	9.00E-03		**5540**	1.05E-02
**2358**	1.90E-02		**7106**	1.14E-02
**6549**	2.00E-02		**5874**	1.96E-02
**7485**	2.50E-02		**4419**	2.22E-02
**7401**	2.90E-02		**4184**	2.36E-02
**6048**	3.50E-02		**5862**	2.57E-02
			**5806**	3.18E-02
			**7939**	3.52E-02
			**4066**	3.56E-02
			**5004**	4.09E-02
			**5919**	4.81E-02
			**3242**	4.99E-02

Peaks with p-value < 0.05

### Identification of the biomarkers

As direct biomarker identification is sensitive with the MALDI apparatus we used, MALDI–MS and FT-ICR-MS parallel analyses were done (see details in methods). C8 and C18 eluats from ALS patients and healthy controls were independently analyzed by HPLC MALDI/FT. In this way, for markers that fit with several HPLC signals (same m/z but different RT), the final selections for identification were based on ALS/healthy HPLC profiles comparison (both, MALDI and FT). By doing so, all peaks of the SVM-based models have been identified (Table 6). Mascot scores and annotated MS/MS spectra are available as supplementary information (Table S4 and Figure S1). It turns out that common m/z between C8 and C18 marker lists (1101 and 1426) correspond to the same peptides. Three of the 7 identified signals are the result of protein degradation and the others are mature small proteins. All of these biomarkers are up-regulated in the plasma of ALS patients, except the peptide derived from the C-terminal part of the complement C3f fragment (C3f), a peptide liberated during the degradation of complement C3d fragment (C3b). The two other identified fragments are stemming on one hand from the extracellular part of the integrin alpha-IIb (ITGA2B), a membrane protein implicated in the platelet system; and on the other hand from the N-terminal part of the Zyxin (ZYX), a cytosolic protein implicated in cell adhesion. The other identified biomarkers are two mature proteins. The first is a cytosolic protein, the thymosin beta-4 (TMSB4X) with an acetylserine at its N-terminal part and the second is a secreted protein, the platelet factor 4 (PF4) detected as both mono- and di-charged signals. The last identified signal is surprisingly a particular form of the PF4 with 4 additional amino acids at the N-terminal part of the protein.

**Table 6:** List of identified peaks.

**m/z**	**Uniprot^1^**	**Protein name**	**Sequence**	**Modification / Comment**
**1101**	P01024	Complement C3	(I)HWESASLLR(S)	C-term fragment [1312-1320] of complement C3f
**1426**	P08514	Integrin alpha-IIb	(R)QIFLPEPEQPSR(L)	Fragment [891-902] with Pyro-Glu (N-term)
**1769**	Q15942	Zyxin	(M)AAPRPSPAISVSVSAPAF(Y)	Fragment [2-19] with N-acetylalanine
**3883**	P02776	Platelet factor 4	(A)EAEEDGDLQCLCVKTTSQVRPRHITSLEVIKAGPHCPTAQLIATLKNGRKICLDLQAPLYKKIIKKLLES	Mature form, (M+2H)^2+^
**4964**	P62328	Thymosin beta-4	(M)SDKPDMAEIEKFDKSKLKKTETQEKNPLPSKETIEQEKQAGES	mature form with N-acetylserine
**7765**	P02776	Platelet factor 4	(A)EAEEDGDLQCLCVKTTSQVRPRHITSLEVIKAGPHCPTAQLIATLKNGRKICLDLQAPLYKKIIKKLLES	mature form
**8141**	P02776	Platelet factor 4	(A)FASAEAEEDGDLQCLCVKTTSQVRPRHITSLEVIKAGPHCPTAQLIATLKNGRKICLDLQAPLYKKIIKKLLES	Fragment [28-101], mature form with 4 additional aa (N-term)

Amino acid in parenthesis: amino acid before or after the identified peptide

^1^ Entry from the Uniprot Knowledgebase database

## Discussion

The diagnosis of ALS is based on the combination of clinical findings in combination with neurophysiologic examinations [1-14]. Consequently, identification of biomarkers for ALS would be important for an early diagnosis. Recent studies have identified individual proteins and/or protein panels from blood plasma and CSF that represent putative biomarkers for ALS, but many of these proteins are not specific of the disease and both sensitivity and specificity of these individual biomarkers remain unsatisfactory [2]. In consequence, discovering a diagnostic using a body fluid necessarily means seeking for a combination of biomarkers to reach satisfactory sensitivity and specificity.

Despite the fact that the CSF is flagged as the most promising body fluid for biomarker research in neurodegenerative diseases, it is also less easy to obtain than blood. Moreover, blood will appear more appropriate in case of a clinical study especially if the goal is to explore the effect of a drug. From blood sample, serum or plasma and PBMC can be obtained. Among these three different sources of biomarkers, plasma is the easiest and the fastest to obtain and theoretically more representative of the physiological reality of a non-coagulated blood. For these different reasons, our study was done using EDTA-plasma samples as recommended in the HUPO plasma proteome project [15].

Among the different methods that can be used to biomarker research in body fluids, mass spectrometry is particularly interesting due to its potential for unbiased screening of samples and for biomarker identification. Some years ago, Villanueva et al. discriminated patients with 3 types of cancers and controls without tumor using peptide extraction by hydrophobic solid phase and MALDI-TOF mass spectrometry read-out [8-16]. With this approach, the low mass range of the blood proteome, particularly peptides with a molecular mass centered around 3,000 Da, could be the source of potential relevant biomarkers. 

In our study, peptide enrichment from ALS and control plasma (40 of each) was done with magnetic beads grafted with two different hydrophobic phases, C8 or C18. In both cases, MALDI profiles from ALS plasma appeared clearly different to age-matched controls spectra. Unsupervised statistical analysis of the MALDI data resulted in distinct patterns that differentiate ALS from controls, with a better ALS/controls differentiation from C18 data than from C8 data. Orientated statistical analyses (SVM) lead to the selection of the most significant peaks and to the working-out of two predictive models based on 2 and 7 peaks for C18- and C8-beads sample preparation respectively. Both these SVM-based models classify blinded samples (validation sets) with very high sensitivities and specificities (greater than 98%). 

The orientated statistical analyses (PLS) of ALS patients according to the ALS subtype shows that spinal and bulbar subtypes can be differentiated on the bases of the C18 MALDI data. Many peaks are involved in this differentiation but only 7 have low p-values (<0.05) and none belong to the final ALS peptide signature previously characterized. These results demonstrated that peptide signatures could be used to discriminate between ALS and healthy volunteers independently of the ALS subtype or the gender of the patients. 

According to the literature blood peptide signatures identified with this magnetic beads-based MALDI-TOF-MS approach correspond to some major proteins of the plasma/serum and/or to fragments of these proteins [17]. Three of the identified biomarkers are actually part of the plasma degradome and the others are mature small proteins of the plasma. For instance, the most down regulated signal in ALS patients has been identified as a fragment of the complement C3f peptide. Derivative peptides from complement C3f have already been flagged as potential biomarkers in different pathologies [18-20]. But mostly characterized peptides were derived from the C3f-desArg peptide which is not the case of the truncated peptide identified in this study (m/z 1101 HWESASLLR, Table 6). To our knowledge this [1312-1320] fragment of the complement C3f was mentioned only one time as potential biomarker in a study of biomarker discovery in liver and inflammatory bowel diseases [21]. But unlike results obtained from Crohn’s disease patients this peptide is down regulated in ALS patients compared to healthy donors. The two other identified fragments were stemming from plasma proteins which have been almost never cited as potential biomarkers from plasma samples, the zyxin and the integrin alpha-IIb [19,22] and they were never flagged as potential peptide biomarkers until now. Moreover it is interesting to note that the integrin alpha-IIb is a marker of platelets whose role in inflammation is known [23]. The remaining identified biomarkers correspond to the mature forms of small plasma proteins also linked to the platelet system, the thymosin beta-4 which plays a vital role in the repair and regeneration of injured cells and the platelet factor 4 (PF-4), a chemokine released during platelet aggregation. Both of these two proteins have already been classified as potential biomarkers, for example in pancreatic cancer [24]. The last identified biomarker is an isoform of PF-4 with 4 additional amino acids at the N-terminal part of the sequence (up-regulated in ALS patients). N-terminally isoforms of a variant of PF4 were characterized by Struyf et al. in 2004 [25], and more recently, seven N-terminally processed PF4 isoforms were identified in serum from healthy volunteers, including the isoform identified in our study [26]. To our knowledge, the isoform of PF4 identified in our study had never been mentioned as potential biomarker in any published study. PF4 and its variant form (PF4V1) are involved in numerous biological processes including inflammation and angiogenesis [27-29]; two processes implicated in neurodegenerative disease such as ALS [30,31]. Increased permeability of the blood-spinal cord-barrier (BSCB) is implicated in the pathogenesis of ALS [32] and haemodynamic mechanism was proposed as a possible cause of the disease [33]. A hypothesis is that the PF4 isoform identified as biomarker of ALS disease might be a marker of one of these phenomena. However, the possible biological role this isoform remains to be investigated in particular in the context of the ALS disease.

All these peptides or small proteins are naturally not direct biomarkers of the ALS disease but can be considered as surrogate biomarkers arising from endogenous protease activities. However, it is of interest that 5 of the 7 identified peaks correspond to proteins of the platelet system (PF4, Integrin alpha II and Thymosin beta-4).The implication of platelets in neurodegenerative diseases (NDDs) such as amyotrophic lateral sclerosis is a phenomenon already reported through several articles [34-36]. Consequently it would be interesting to analyze these markers in other neurodegenerative diseases to evaluate their capability to discriminate ALS patients to patients suffering of others NDDs. 

In summary, we have constructed two diagnostic models for ALS based on a combination of biomarkers using MALDI-TOF-MS profiling combined with C18 and C8 magnetic bead peptide enrichment. Patients with ALS can be effectively distinguished from age-matched healthy patients using these two predictive models with very high sensitivities and specificities. 

## Supporting Information

Figure S1 **Biomarker identification by nanoLC-MS/MS.** MSMS Spectrum of the m/z MALDI peaks 1101 (A), 1426 (B), 1769 (C), 4964 (D), 7765 (E) and 8141 (F).
(PDF)

Table S1 **Matrix of C8 detected peaks.** (XLS)

Table S2 **Matrix of C18 detected peaks.** (XLS)

Table S3 **List of the up- or down-regulated peaks in ALS patients.** A/ C8-peaks, B/ C18-peaks (spectrum data were normalized with loess and quantile normalization).
(PDF)

Table S4 **Mascot search results for the identified biomarkers.** (PDF)
