# Supplementary material for: Plasma Peptide Biomarker Discovery for Amyotrophic Lateral Sclerosis by MALDI –TOF Mass Spectrometry Profiling
Source: PLoS One. 2013 Nov 5;8(11):e79733. doi: 10.1371/journal.pone.0079733 (PMC3818176; doi:10.1371/journal.pone.0079733)
Supplement: Figure S1 — Biomarker identification by nanoLC-MS/MS. MSMS Spectrum of the m/z MALDI peaks 1101 (A), 1426 (B), 1769 (C), 4964 (D), 7765 (E) and 8141 (F). (PDF) [file pone.0079733.s001.pdf]

Figure S1. Biomarker identification by nanoLC-MS/MS: MSMS Spectrum of the m/z MALDI peaks 1101 (A), 1426 (B), 1769 (C), 4964 (D), 7765 (E) and 8141 (F)

A **m/z 1101**

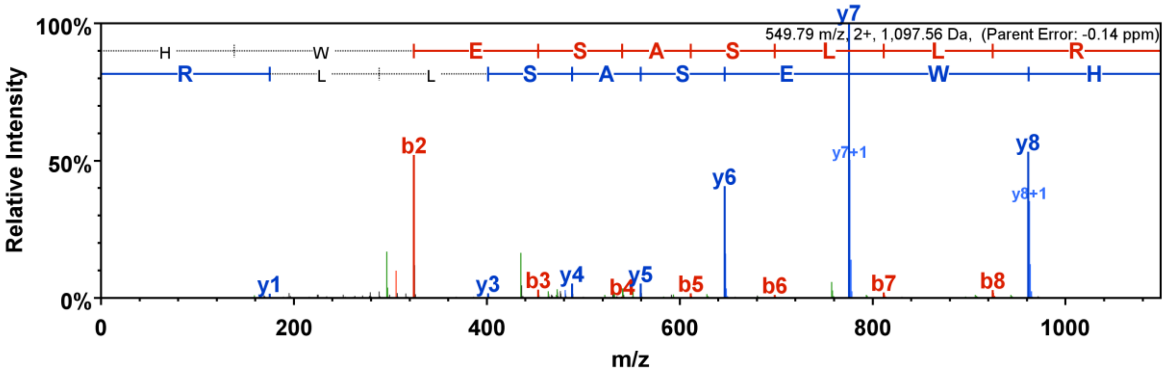

B **m/z 1426**

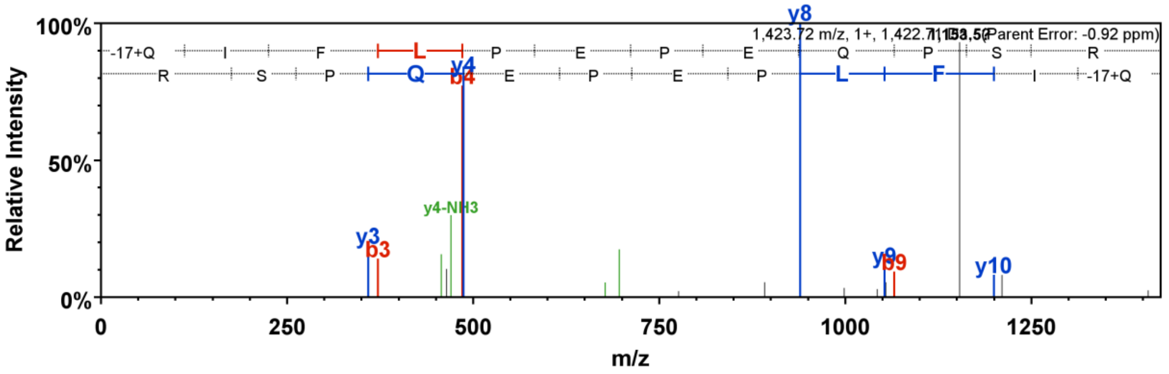

C **m/z 1769**

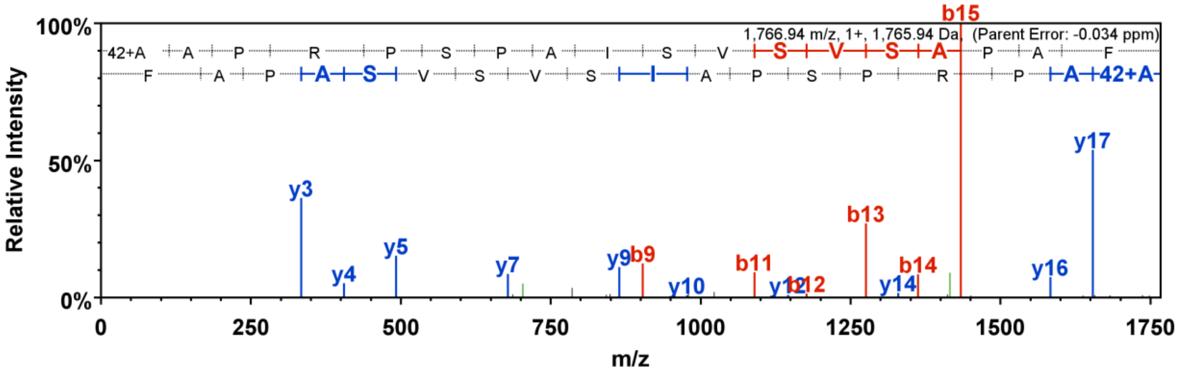

D

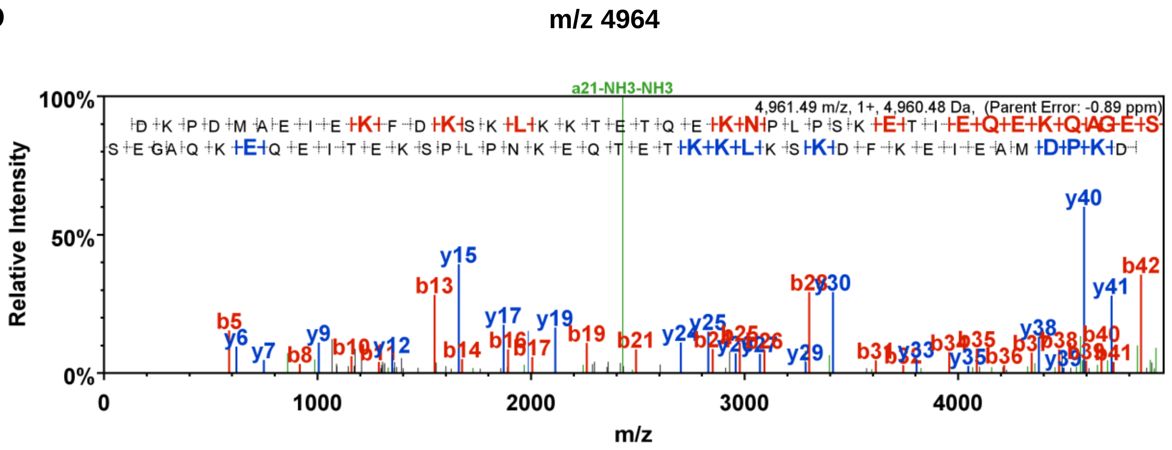
m/z 4964
4,961.49 m/z, 1+, 4,960.48 Da, (Parent Error: -0.89 ppm)
Relative Intensity
100%
50%
0%
m/z
0
1000
2000
3000
4000
a21-NH3-NH3
y40
b42
y15
b13
y30
y41
y17
y19
y25
y38
b40
b5
y6
y7
y9
b8
y12
b14
b16
b17
b19
b21
y24
b26
y29
b31
y33
b35
b36
y39
b41

E

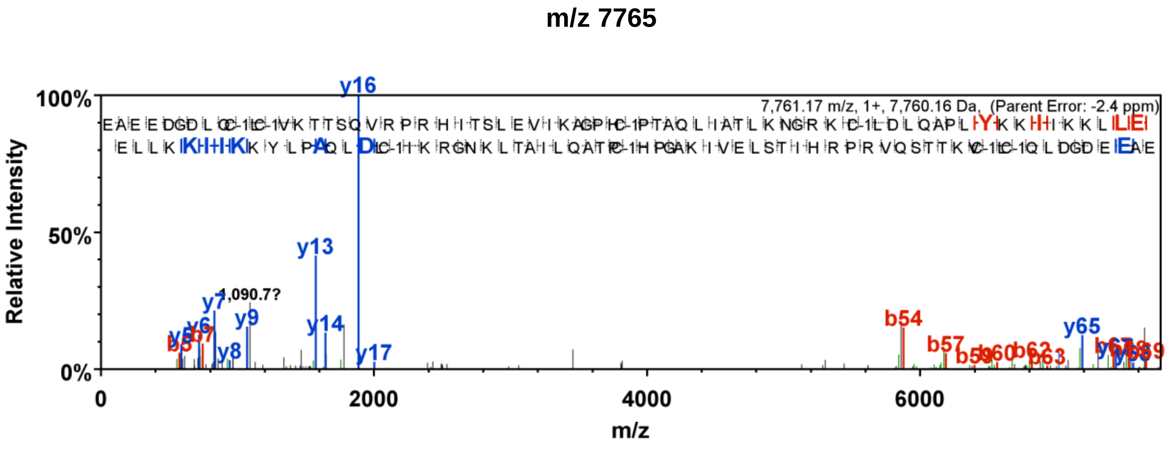
m/z 7765
7,761.17 m/z, 1+, 7,760.16 Da, (Parent Error: -2.4 ppm)
Relative Intensity
100%
50%
0%
m/z
0
2000
4000
6000
y16
y13
4,090.7?
y7
y9
y6
y8
y14
y17
b54
b57
b62
b63
y65

F

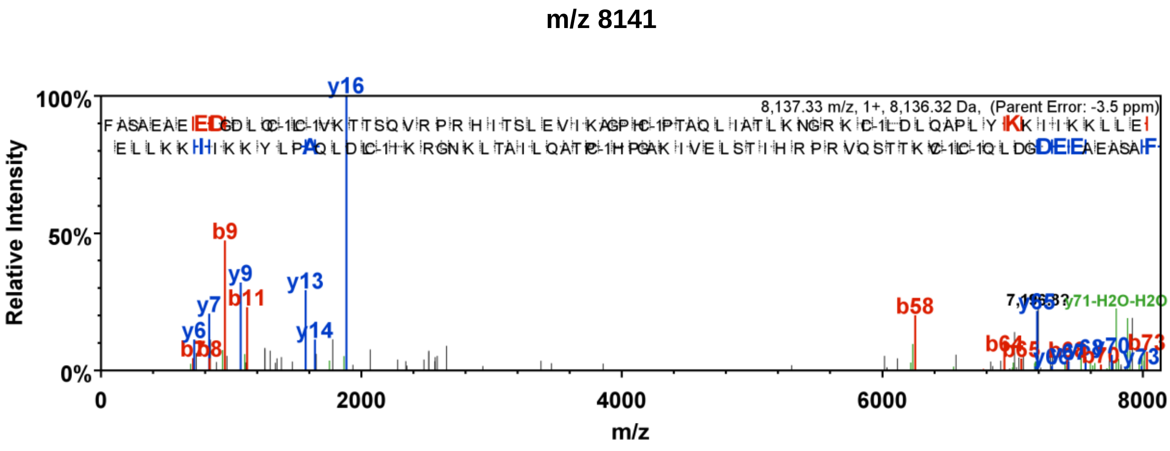
m/z 8141
8,137.33 m/z, 1+, 8,136.32 Da, (Parent Error: -3.5 ppm)
Relative Intensity
100%
50%
0%
m/z
0
2000
4000
6000
8000
y16
b9
y9
y13
b11
y7
y6
y14
b8
b58
y71-H2O-H2O
b64
b73
y73
