# Supplementary material for: Plasma Peptide Biomarker Discovery for Amyotrophic Lateral Sclerosis by MALDI –TOF Mass Spectrometry Profiling
Source: PLoS One. 2013 Nov 5;8(11):e79733. doi: 10.1371/journal.pone.0079733 (PMC3818176; doi:10.1371/journal.pone.0079733)
Supplement: Table S3 — List of the up- or down-regulated peaks in ALS patients. A/ C8-peaks, B/ C18-peaks (spectrum data were normalized with loess and quantile normalization). (PDF) [file pone.0079733.s004.pdf]

Table S3. List of the up- or down-regulated peaks in ALS patients

A/ C8-peaks, B/ C18-peaks (spectrum data were normalized with loess and quantile normalization)

A/ C8-peaks							
Up ALS	Log Effect Size	Effect Size	P-Value	Down ALS	Log Effect Size	Effect Size	P-Value
7765	1.5841	2.9982	1.81E-138	1101	-0.5594	0.6786	3.00E-45
4964	0.7870	1.7254	1.73E-104	8765	-0.5209	0.6969	1.35E-28
8141	0.7332	1.6623	1.86E-98	4382	-0.4172	0.7489	2.67E-27
3883	0.8993	1.8651	6.41E-86	1127	-0.3448	0.7874	3.35E-21
9288	0.7595	1.6929	2.97E-76	1898	-0.5213	0.6968	8.67E-18
1769	0.6010	1.5168	6.85E-69	1881	-0.2797	0.8238	1.16E-16
6630	0.8379	1.7875	1.28E-53	9379	-0.3076	0.8080	1.54E-14
7839	0.3847	1.3056	1.91E-44	2569	-0.1879	0.8779	2.58E-13
6432	0.7723	1.7080	3.24E-42	1924	-0.2467	0.8428	3.58E-13
3316	0.5482	1.4623	3.42E-42	4566	-0.2251	0.8556	4.87E-13
4644	0.4291	1.3464	3.68E-42	4690	-0.2635	0.8331	1.62E-12
6588	0.5655	1.4799	4.90E-39	4361	-0.2282	0.8537	2.81E-12
3217	0.3647	1.2877	7.01E-37	8724	-0.1836	0.8805	5.13E-12
4071	0.2169	1.1622	6.91E-23	1044	-0.2259	0.8550	2.43E-11
6530	0.2764	1.2112	1.22E-19	4885	-0.1294	0.9142	1.81E-10
1426	0.2478	1.1874	1.05E-16	4102	-0.1351	0.9106	1.08E-09
6389	0.2133	1.1594	3.20E-13	1214	-0.1874	0.8782	1.23E-09
8564	0.1917	1.1421	2.52E-11	1084	-0.2428	0.8451	1.40E-09
7639	0.1628	1.1195	7.75E-10	7397	-0.1316	0.9128	1.52E-09
6731	0.1301	1.0944	1.78E-07	9485	-0.2801	0.8236	4.95E-09
6715	0.1248	1.0903	5.37E-07	1465	-0.1794	0.8831	9.24E-09
3295	0.1369	1.0995	7.90E-07	9571	-0.1526	0.8996	3.30E-08
7933	0.1663	1.1222	1.42E-05	8307	-0.1448	0.9045	3.89E-08
6327	0.0952	1.0682	7.58E-05	2755	-0.3880	0.7642	4.34E-08
6479	0.1183	1.0855	2.41E-04	1713	-0.1324	0.9123	1.57E-07
7649	0.0970	1.0695	2.91E-04	2742	-0.1698	0.8890	1.98E-07
6369	0.0779	1.0555	8.02E-04	3044	-0.1006	0.9326	9.18E-07
3903	0.0848	1.0605	1.85E-03	1742	-0.1384	0.9085	9.64E-07
1867	0.0786	1.0560	5.54E-03	9132	-0.1158	0.9228	3.00E-06
4282	0.0649	1.0460	9.61E-03	7345	-0.0993	0.9335	3.37E-06
8101	0.0512	1.0361	1.22E-02	1027	-0.2370	0.8485	5.33E-06
6775	0.0418	1.0294	5.54E-02	4404	-0.1075	0.9282	7.44E-06
6921	0.0471	1.0332	9.81E-02	7469	-0.0972	0.9348	9.05E-06
1382	0.0453	1.0319	1.20E-01	7110	-0.0939	0.9370	9.61E-06
3340	0.0316	1.0221	2.33E-01	1068	-0.1978	0.8719	9.66E-06
4435	0.0270	1.0189	2.72E-01	4481	-0.1442	0.9049	1.13E-05

6801	0.0238	1.0167	2.83E-01
8810	0.0589	1.0417	2.91E-01
7191	0.0156	1.0109	4.82E-01
7564	0.0303	1.0212	5.36E-01
3430	0.0199	1.0139	5.60E-01
3475	0.0147	1.0102	5.63E-01
3743	0.0081	1.0056	7.99E-01
5172	0.0055	1.0038	8.15E-01

6968	-0.1075	0.9282	1.19E-05
2248	-0.1781	0.8839	1.23E-05
8961	-0.2140	0.8621	1.49E-05
2430	-0.0974	0.9347	2.45E-05
1526	-0.1215	0.9192	3.51E-05
4788	-0.0926	0.9379	4.70E-05
1995	-0.1484	0.9023	5.00E-05
8625	-0.1122	0.9252	9.70E-05
8601	-0.1193	0.9206	2.15E-04
2793	-0.0954	0.9360	3.05E-04
9422	-0.1573	0.8967	4.05E-04
5618	-0.0805	0.9457	7.14E-04
9935	-0.1130	0.9246	1.00E-03
6880	-0.1064	0.9289	2.71E-03
3141	-0.0664	0.9550	5.25E-03
5042	-0.0867	0.9417	8.89E-03
4343	-0.0674	0.9543	9.34E-03
8006	-0.0728	0.9508	1.28E-02
7296	-0.0506	0.9655	1.29E-02
4457	-0.1047	0.9300	2.18E-02
5289	-0.0586	0.9602	2.68E-02
2024	-0.0720	0.9513	2.98E-02
9668	-0.0641	0.9565	2.99E-02
5942	-0.0401	0.9726	3.05E-02
8915	-0.0977	0.9345	4.86E-02
4627	-0.0597	0.9594	6.82E-02
8523	-0.0378	0.9742	6.95E-02
2939	-0.0766	0.9483	7.60E-02
7438	-0.0401	0.9726	7.86E-02
8204	-0.0923	0.9380	7.94E-02
4300	-0.0437	0.9702	1.21E-01
4856	-0.0360	0.9753	1.44E-01
2118	-0.0769	0.9481	1.62E-01
5131	-0.0339	0.9767	1.77E-01
2972	-0.0288	0.9802	1.87E-01
9644	-0.0475	0.9676	1.88E-01
5799	-0.0283	0.9806	2.18E-01
9714	-0.0405	0.9723	2.33E-01
7020	-0.0354	0.9758	2.77E-01
6940	-0.0286	0.9803	3.34E-01
5863	-0.0239	0.9836	3.49E-01

2284	-0.0250	0.9828	3.69E-01
3087	-0.0238	0.9837	4.01E-01
8582	-0.0261	0.9821	4.06E-01
5753	-0.0167	0.9885	4.31E-01
2212	-0.0108	0.9926	6.34E-01
7667	-0.0086	0.9940	7.33E-01
7155	-0.0099	0.9932	7.42E-01
5707	-0.0050	0.9966	8.16E-01
8685	-0.0077	0.9947	8.65E-01
8869	-0.0049	0.9966	9.05E-01

B/ C18-peaks							
Up ALS	Log Effect Size	Effect Size	P-Value	Down ALS	Log Effect Size	Effect Size	P-Value
1426	1.4895	2.8080	4.71E-207	1101	-2.0131	0.2477	1.12E-217
2511	1.1752	2.2582	3.94E-182	1127	-1.4864	0.3569	5.50E-132
4979	1.8548	3.6171	5.86E-181	2755	-1.4319	0.3707	6.66E-104
4964	2.3044	4.9396	6.77E-176	2662	-0.9899	0.5035	2.66E-99
2483	1.4045	2.6473	3.51E-174	1784	-0.5439	0.6859	2.01E-70
4920	1.2315	2.3480	5.24E-141	1079	-1.0164	0.4944	3.82E-51
5004	1.1429	2.2082	7.51E-129	7348	-0.5360	0.6897	1.04E-43
3469	1.1271	2.1842	1.27E-123	4795	-0.3813	0.7677	8.52E-43
3366	0.8603	1.8155	1.01E-119	1214	-0.4586	0.7277	3.99E-40
3218	0.8322	1.7804	4.22E-113	3264	-0.3904	0.7629	4.99E-39
3405	0.6588	1.5788	1.01E-100	1013	-0.6218	0.6498	6.06E-38
4426	1.0933	2.1336	2.72E-100	1854	-0.3545	0.7821	5.30E-37
2230	1.4769	2.7836	3.72E-100	1370	-0.3465	0.7865	2.24E-36
1769	0.7906	1.7298	1.01E-92	2770	-0.4076	0.7539	1.09E-32
4066	0.4861	1.4007	8.07E-68	1157	-0.4080	0.7536	3.96E-31
5109	0.5034	1.4176	1.49E-61	1501	-0.3958	0.7601	1.89E-30
3929	0.3175	1.2461	2.34E-39	1063	-0.8271	0.5637	1.60E-29
4899	0.3768	1.2984	6.64E-38	5905	-0.4633	0.7253	4.44E-29
2194	0.2956	1.2274	7.68E-35	4018	-0.2680	0.8305	1.25E-27
3762	0.3517	1.2761	8.70E-35	1506	-0.4154	0.7498	8.52E-26
4528	0.2827	1.2164	7.68E-32	2380	-0.2770	0.8253	9.75E-26
7558	0.2347	1.1767	3.96E-25	7316	-0.2249	0.8556	2.71E-25
4442	0.4110	1.3296	1.26E-24	1085	-0.6557	0.6348	4.44E-25
4850	0.2493	1.1887	1.25E-22	8763	-0.4302	0.7422	4.75E-25
5248	0.2560	1.1942	5.65E-19	5806	-0.2203	0.8584	4.91E-24
9289	0.7923	1.7318	1.58E-17	2125	-0.3181	0.8021	3.05E-23
1867	0.2879	1.2209	1.83E-17	1452	-0.3019	0.8112	5.52E-23
4384	0.2480	1.1875	6.71E-16	5919	-0.2898	0.8180	3.34E-21

6910	0.1758	1.1296	4.46E-14
7519	0.1608	1.1179	6.20E-14
5133	0.2578	1.1956	3.25E-13
6756	0.1493	1.1090	2.07E-11
7126	0.1448	1.1056	5.12E-11
4645	0.3944	1.3144	1.10E-10
2007	0.1913	1.1418	2.67E-09
6784	0.1340	1.0973	4.11E-09
6879	0.1938	1.1438	1.35E-08
5067	0.1106	1.0797	2.77E-07
7010	0.0970	1.0695	1.70E-06
6174	0.1396	1.1016	1.78E-06
6861	0.1086	1.0782	4.65E-06
7499	0.0839	1.0599	5.21E-05
7485	0.0878	1.0627	6.85E-05
6191	0.0920	1.0658	9.80E-05
8992	0.0901	1.0645	9.87E-05
6455	0.0904	1.0646	1.20E-04
4419	0.2186	1.1636	1.81E-04
6807	0.1071	1.0770	5.75E-04
2023	0.0511	1.0361	2.07E-03
2048	0.1068	1.0768	2.26E-03
6985	0.0679	1.0482	2.67E-03
7062	0.0631	1.0447	5.84E-03
7266	0.0525	1.0371	2.15E-02
7223	0.0458	1.0323	3.18E-02
1742	0.0628	1.0445	4.16E-02
9176	0.0983	1.0705	4.44E-02
7441	0.0385	1.0270	4.73E-02
6549	0.0509	1.0359	4.82E-02
7241	0.0326	1.0228	1.03E-01
8105	0.0359	1.0252	1.27E-01
8142	0.0335	1.0235	1.92E-01
2863	0.0599	1.0424	2.07E-01
2358	0.0721	1.0512	2.36E-01
7106	0.0179	1.0125	3.55E-01
7766	0.0548	1.0387	4.23E-01
7727	0.0190	1.0132	4.58E-01
6386	0.0073	1.0051	7.82E-01

1937	-0.3261	0.7977	4.22E-21
5862	-0.2906	0.8176	6.01E-21
1288	-0.3802	0.7684	2.21E-19
1882	-0.3291	0.7960	6.42E-19
4250	-0.2761	0.8258	8.68E-19
8933	-0.2153	0.8614	4.79E-18
3280	-0.1984	0.8715	6.27E-18
8705	-0.2747	0.8266	6.92E-18
1980	-0.2694	0.8297	7.78E-18
6242	-0.2119	0.8634	8.19E-15
8917	-0.1803	0.8825	8.97E-15
4102	-0.2140	0.8621	1.36E-14
2933	-0.3612	0.7785	3.07E-13
4184	-0.1810	0.8821	1.04E-12
3770	-0.1855	0.8794	1.43E-12
5874	-0.2414	0.8459	7.08E-12
1608	-0.2255	0.8553	8.22E-12
5844	-0.1957	0.8731	1.09E-11
8436	-0.2875	0.8193	3.39E-11
8008	-0.1299	0.9139	6.93E-11
7663	-0.1344	0.9111	8.44E-10
7850	-0.1449	0.9045	9.89E-10
4679	-0.1468	0.9032	1.09E-09
4373	-0.1910	0.8760	2.09E-09
8353	-0.1780	0.8839	3.05E-09
8273	-0.1102	0.9264	1.59E-07
3193	-0.1295	0.9141	2.93E-07
6047	-0.1236	0.9179	4.07E-07
1924	-0.2655	0.8319	9.61E-07
9134	-0.2961	0.8144	1.79E-06
1898	-0.1014	0.9321	5.89E-06
8052	-0.0903	0.9393	6.41E-06
7924	-0.0872	0.9413	7.12E-06
5589	-0.1095	0.9269	7.28E-06
7939	-0.0929	0.9376	1.94E-05
8512	-0.2566	0.8371	2.24E-05
3884	-0.1315	0.9129	2.61E-05
4575	-0.2736	0.8272	1.00E-04
8864	-0.0861	0.9421	1.98E-04
9484	-0.1363	0.9099	4.07E-04
6090	-0.0858	0.9423	5.84E-04

9426	-0.1568	0.8970	1.56E-03
9462	-0.0823	0.9446	1.88E-03
1468	-0.1717	0.8878	2.02E-03
8234	-0.0551	0.9625	1.08E-02
4302	-0.1311	0.9131	1.54E-02
2063	-0.0785	0.9470	1.87E-02
5668	-0.0620	0.9580	2.39E-02
7637	-0.0485	0.9669	2.62E-02
6630	-0.0525	0.9642	2.64E-02
2792	-0.0954	0.9360	5.30E-02
9391	-0.0547	0.9628	1.18E-01
7422	-0.0333	0.9772	1.29E-01
5945	-0.0403	0.9724	1.43E-01
7401	-0.0267	0.9816	2.38E-01
3242	-0.0755	0.9490	2.54E-01
4590	-0.0486	0.9668	2.55E-01
9063	-0.0402	0.9725	2.78E-01
5540	-0.0259	0.9822	3.01E-01
8603	-0.0391	0.9733	5.76E-01
7609	-0.0080	0.9945	7.00E-01
6950	-0.0091	0.9937	7.74E-01
2954	-0.0039	0.9973	8.89E-01
