# Supplementary material for: Plasma Peptide Biomarker Discovery for Amyotrophic Lateral Sclerosis by MALDI –TOF Mass Spectrometry Profiling
Source: PLoS One. 2013 Nov 5;8(11):e79733. doi: 10.1371/journal.pone.0079733 (PMC3818176; doi:10.1371/journal.pone.0079733)
Supplement: Table S4 — Mascot search results for the identified biomarkers. (PDF) [file pone.0079733.s005.pdf]

Table S4: Mascot search results for the identified biomarkers

MALDI m/z	Sequence	Acces Nb: Protein name	Prob	Mascot Ion score	Mascot Identity score	Mascot Delta Ion Score	Modifications	Observed	Actual Mass	Delta PPM
1101	(I)HWESASLLR(S)	P01024: Complement C3	100%	46.18	41.36	35.61		549.7881	1,097.56	-0.1365
1426	(R)qIFLPEPEQPSR(L)	P08514: Integrin alpha-IIb	100%	27.25	43.08	17.67	Gln->pyro-Glu (-17)	1,423.72	1,422.71	-0.9212
1769	(M)aAPRPSPAISVSVSAPAF(Y)	Q15942: Zyxin	100%	65.77	42.57	41.42	Acetyl (+42)	1,766.94	1,765.94	-0.03376
4964	(M)sDKPDMAEIEKFDKSKLKKTETQEKNPLPSKETIEQEKQAGES(-)	P62328: Thymosin beta-4	100%	156.96	46.79	156.96	Acetyl (+42)	4,961.49	4,960.48	-0.8924
7765	(A)EAEEDGDLQcLcVKTTSQVRPRHITSLEVIKAGPHcPTAQLIATLKNGRKIcLDLQAPLYKKIIKKLLES(-)	P02776: Platelet factor 4	100%	18.63	47.98	18.63	4 Dehydro (-4)	7,761.17	7,760.16	-2.434
8141	(A)FASAEAEEDGDLQcLcVKTTSQVRPRHITSLEVIKAGPHcPTAQLIATLKNGRKIcLDLQAPLYKKIIKKLLES(-)	P02776: Platelet factor 4	100%	26.31	49.18	26.31	4 Dehydro (-4)	8,137.33	8,136.32	-3.482
